# Explainability of random survival forests in predicting conversion risk from mild cognitive impairment to Alzheimer’s disease

**DOI:** 10.1186/s40708-023-00211-w

**Published:** 2023-11-18

**Authors:** Alessia Sarica, Federica Aracri, Maria Giovanna Bianco, Fulvia Arcuri, Andrea Quattrone, Aldo Quattrone, for the Alzheimer’s Disease Neuroimaging Initiative

**Affiliations:** Neuroscience Research Center, Department of Medical and Surgical Sciences, Magna Graecia University, viale Europa, loc. Germaneto, 88100 Catanzaro, Italy

**Keywords:** Survival analysis, Cox proportional hazard, Random Survival Forests, Machine learning, MCI conversion, AD progression, Dementia risk

## Abstract

Random Survival Forests (RSF) has recently showed better performance than statistical survival methods as Cox proportional hazard (CPH) in predicting conversion risk from mild cognitive impairment (MCI) to Alzheimer’s disease (AD). However, RSF application in real-world clinical setting is still limited due to its black-box nature.

For this reason, we aimed at providing a comprehensive study of RSF explainability with SHapley Additive exPlanations (SHAP) on biomarkers of stable and progressive patients (sMCI and pMCI) from Alzheimer’s Disease Neuroimaging Initiative. We evaluated three global explanations—RSF feature importance, permutation importance and SHAP importance—and we quantitatively compared them with Rank-Biased Overlap (RBO). Moreover, we assessed whether multicollinearity among variables may perturb SHAP outcome. Lastly, we stratified pMCI test patients in high, medium and low risk grade, to investigate individual SHAP explanation of one pMCI patient per risk group.

We confirmed that RSF had higher accuracy (0.890) than CPH (0.819), and its stability and robustness was demonstrated by high overlap (RBO > 90%) between feature rankings within first eight features. SHAP local explanations with and without correlated variables had no substantial difference, showing that multicollinearity did not alter the model. FDG, ABETA42 and HCI were the first important features in global explanations, with the highest contribution also in local explanation. FAQ, mPACCdigit, mPACCtrailsB and RAVLT immediate had the highest influence among all clinical and neuropsychological assessments in increasing progression risk, as particularly evident in pMCI patients’ individual explanation. In conclusion, our findings suggest that RSF represents a useful tool to support clinicians in estimating conversion-to-AD risk and that SHAP explainer boosts its clinical utility with intelligible and interpretable individual outcomes that highlights key features associated with AD prognosis.

**Graphical Abstract:**

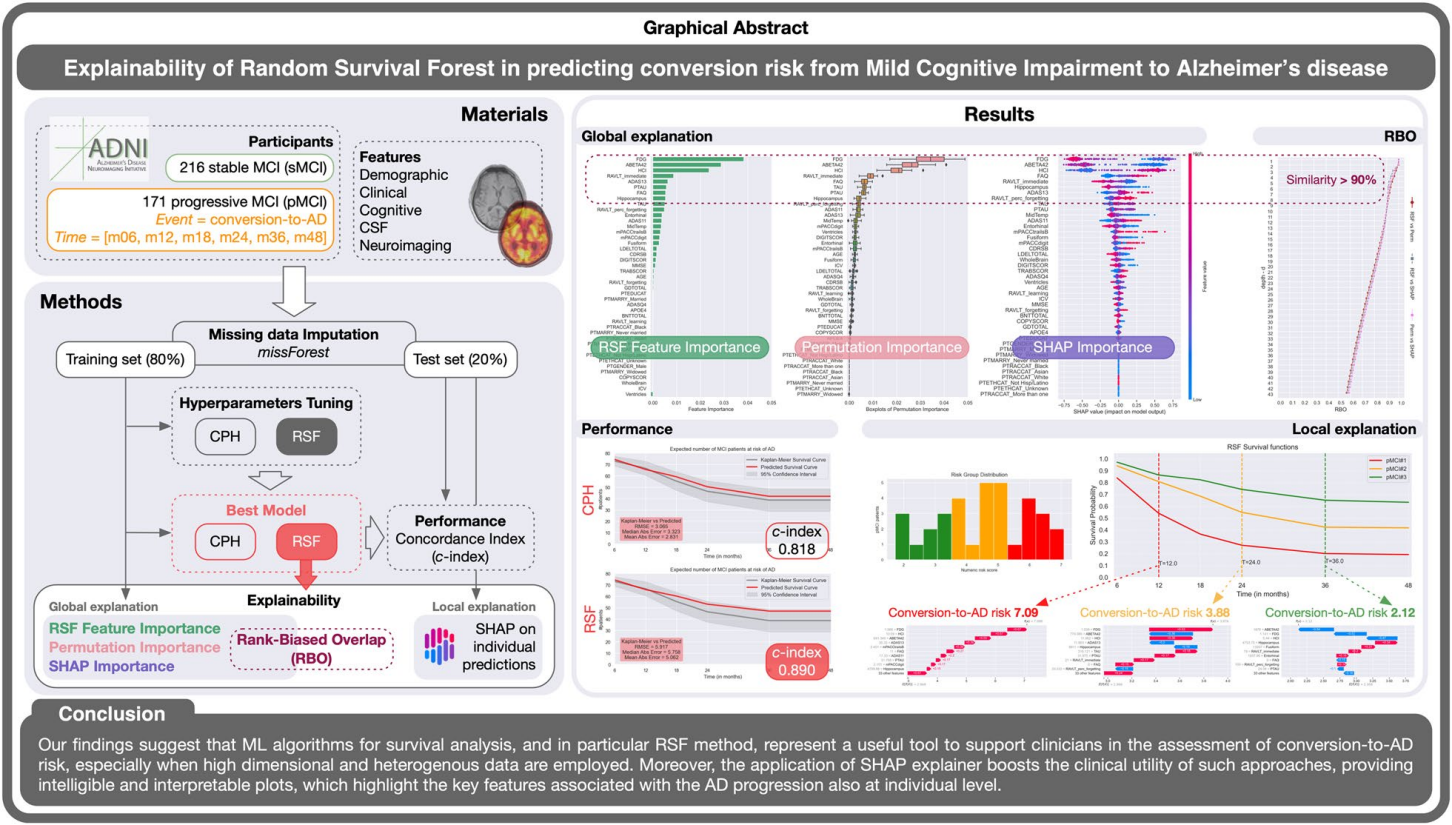

**Supplementary Information:**

The online version contains supplementary material available at 10.1186/s40708-023-00211-w.

## Introduction

Alzheimer’s disease (AD) is the most common form of dementia among the elderly, representing 60–70% of the cases worldwide [1]. The diagnosis of AD consists in a complex assessment of clinical, neuropsychological, cerebrospinal fluid (CSF) biomarkers and neuroimaging information [2]. The complexity in AD diagnosis increases at early stages, because symptoms could fall between normal aging changes and early dementia [1]. It has been estimated that patients affected by Mild Cognitive Impairment (MCI), which is a heterogeneous condition characterized by subjective cognitive complaints, have a 33.6% cumulative risk to progress to AD [3]. For this reason, early prediction of conversion from MCI to AD is crucial for the management of a successful medical treatment.

Artificial Intelligence (AI) and Machine Learning (ML) reached excellent accuracy for the early diagnosis of AD [4–6] and for the prediction of progression from MCI to AD [5, 7, 8], showing also good interpretability and explainability [9, 10]. ML algorithms used with these aims are supervised learning approaches—typically binary classifiers—which are trained on multi-modal data to distinguish between stable MCI patients (sMCI) and progressive MCI patients (pMCI), who change their diagnosis to AD over time [4, 9, 11]. Together with clinical scales, cognitive assessment and neuropsychological tests [12], data from neuroimaging, such as magnetic resonance imaging (MRI) [13], fluorodeoxyglucose (FDG)-positron emission tomography (PET) [14], and FDG-derived hypometabolic convergence index [15] (HCI), resulted to be accurate biomarkers to predict AD at different prodromal stages. Aggregation of amyloid-β into amyloid plaques (Aβ40 and Aβ42) and of tau into neurofibrillary tangles (total tau and phosphorylated tau, *p*-tau) are two CSF biomarkers typically linked to AD [16]. Genetic risk factors represent other important biomarkers associated to AD, for example APOE-ε 4 allele accounts for 20–25% of cases [17]. All these biomarkers had shown high predictive power when used to train ML classifiers, but the weakness of classical supervised algorithms is that they could not handle the time-to-AD conversion and they do not provide any evaluation of the progression risk from MCI to AD. Most importantly, ML classification is not capable of handling right-censored datasets in which the event of interest is not observed for some subjects before the study is terminated [18].

Survival analysis is a statistic field that was born to predict the time-to-event in presence of right censoring [18]. Cox proportional hazard [19] (CPH) is widely applied in survival studies, but it is able to deal only with small datasets and it does not scale well to high-dimensional feature space [20]. To overcome CPH weaknesses, ML algorithms were adapted to handle censored data so as to predict the time-to-event on high-dimensional and heterogeneous data [20, 21] with optimal performance. Among novel ML survival methods, those based on decision trees and in particular on Random Forests (RF) [4, 22] provided promising results on biomedical dataset [23, 24]. The strength of tree-based survival models relies on their independence from data distribution since they are fully nonparametric, on their capability of handling multicollinearity, and on their intrinsic feature selection [25]. In a very recent work [26], we compared the performance of three RF-based survival methods, Random Survival Forests (RSF) [27], Conditional Survival Forest (CSF) [28] and Extra Survival Trees (XST) [29], in predicting the conversion-to-AD risk on dementia biomarkers from the Alzheimer’s Disease Neuroimaging Initiative (ADNI). We found that RSF had the best prediction accuracy compared with CSF and XST, as well as with CPH, thanks to its important characteristics: robustness to outliers, absence of convergence issues, out-of-bag (cross-validated) prediction that ensures no overfitting, reliable inference of training data, and lastly its fully nonparametric variable importance measure of features’ contribution to predict survival function [27]. Although RSF demonstrated optimal performance in the prediction of conversion-to-AD risk in several works [20, 26, 30, 31], its application in a real-world clinical setting to assist prognosis is still limited due to its black-box nature, which results in poorly explainable and interpretable outcomes. Explainable Artificial Intelligence (XAI) and interpretable ML [32–34] provide solutions to this issue [9, 25, 35, 36], trying to unveil the black-box through model-agnostic methods like Local Interpretable Model-Agnostic Explanations (LIME) [37] and SHapley Additive exPlanations (SHAP) [38].

Although LIME and SHAP are usually applied for classification problems, SHAP was recently used also for the investigation of ML survival analysis methods, as for example in a breast cancer survival study [39], and in works for the survival prediction of anaplastic thyroid carcinoma [40] and of heart failure [41], but it was never used for the survival analysis of dementia. For this reason, in the present study we applied SHAP method to investigate both global and local explanations of RSF in the prediction of conversion-to-AD risk within 4 years. We used CPH as benchmark for performance and we increased the feature space of our recent work [26] with further well-known biomarkers from ADNI. First, we provided an overall analysis of RSF variable importance in comparison with permutation importance [42] and SHAP feature importance, to investigate the stability and robustness of the survival model on training set. We quantitatively compared these three variable rankings through the Rank-Biased Overlap (RBO) [43], a similarity measure between rankings that has been employed also to estimate the percentage overlap between feature importance [35]. We applied an automatic variable selection on the three different importance measures to confirm or reject recent literature that revealed no improvement in survival methods performance when feature selection is applied [23, 39]. As further analysis, we investigated whether multicollinearity among variables may perturb the explanations and with this aim we built two SHAP explainers with and without correlated features. Finally, we stratified pMCI test subjects in three risk grades—high, medium and low—according to the RSF predicted risk score and we explored the SHAP local explanations of one pMCI patient per risk grade together with one sMCI patient.

## Related works

Survival analysis with ML algorithms is a relatively novel field and very few works investigated its application on dementia data for predicting conversion risk from MCI to AD [20, 21, 30, 31, 44, 45]. Orozco-Sanchez et al. [21] proposed a unified approach for the study of ML Cox models applied on MCI patients data from the Alzheimer’s Disease Neuroimaging Initiative (ADNI) database, with more than 300 quantitative MRI (qMRI) features. They trained four Cox regression models with different strategies for feature selection. The best model was the Penalized Cox Regression (Coxnet), which reached a *c*-index of 0.84 (95% CI 0.82–0.86).

Performance of Cox model was compared with a deep learning-based (DeepHit) method by Nakagawa et al. [44] on brain gray matter volumes of MCI patients from ADNI database. Their proposed model consisted in a deep neural network based on a Weibull distribution, which achieved a concordance index of 0.835, higher than the value 0.75 of the traditional standard Cox proportional hazard model.

Spooner et al. [20] performed a survival analysis for the prediction of conversion-to-AD risk on two dementia datasets, the Sydney Memory and Ageing Study (MAS) and the ADNI. The feature space consisted in demographics, genetic data, cognitive assessments, neuropsychological scores and other heterogenous information. They compared the prediction performance of ten ML survival approaches on such high-dimensional datasets, and they found that best accuracy on MAS and ADNI were, respectively, 0.82 with a Cox model with likelihood-based boosting and 0.93 with an ElasticNet, while the penalized Cox regression model had the worst performance on both datasets.

In the work of Mirabnahrazam et al. [45], a deep learning-based survival model (DeepSurv) was applied to estimate the time-to-conversion to AD on ADNI data, including demographics, cognitive tests, genetic data, cerebrospinal fluid biomarker and MRI measures. DeepSurv is a model that extends the classic CPH, and it showed an accuracy of 0.831 on a subset of most important features.

In a very recent work, Musto et al. [30] compared the performance of Survival Random Forest (SRF) CPH and Survival Deep Hit Neural Networks (SNN), in predicting time-to-AD diagnosis on heterogenous data from ADNI, such as demographics, MRI, CSF and PET data. They demonstrated the superiority of SRF, which had on MCI patients an accuracy of 0.84, while CPH and Deep Hit reached, respectively, 0.78 and 0.83. The optimal performance of Random Survival Forests (RSF) was also demonstrated in another very recent work by Song et al. [31], which used two dementia cohorts, the National Alzheimer Coordinating Center (NACC) and ADNI, with six predictors: delayed logical memory score (story recall), CDR Dementia Staging Instrument—Sum of Boxes, general orientation in CDR, ability to remember dates and ability to pay bills in the Functional Activities Questionnaire, and patient age. The accuracies of the model were 90.82% and 86.51% in NACC and ADNI, respectively.

Finally, our previous work by Sarica et al. [26], demonstrated that RSF had better performance (0.87) than other two tree-based survival algorithms, Conditional Survival Forest and Extra Survival Trees (both 0.85), and than CPH (0.83) in predicting conversion-to-AD risk on ADNI dataset.

## Materials and methods

### Dataset preparation

Data used in the preparation of this article were obtained from the Alzheimer’s Disease Neuroimaging Initiative (ADNI) database (adni.loni.usc.edu). The ADNI was launched in 2003 as a public–private partnership, led by Principal Investigator Michael W. Weiner, MD. The primary goal of ADNI has been to test whether serial magnetic resonance imaging (MRI), positron emission tomography (PET), other biological markers, and clinical and neuropsychological assessment can be combined to measure the progression of mild cognitive impairment (MCI) and early Alzheimer’s disease (AD).

In detail, for the preparation of the dataset, two main table files (*csv*) from ADNI were used: DXSUM_PDXCONV_ADNIALL, which contains the information about the diagnosis conversion (e.g., from MCI to AD or other kind of dementia), and ADNIMERGE, which contains demographic, clinical, cognitive, and imaging data of patients. Other table files used are: NEUROBAT, CDR, GDSCALE, FAQ, MMSE, ADASSCORES, UPENNBIOMK_MASTER_FINAL (9, 10 12), BAIPETNMRC_04_12_18. All files were downloaded the 5^th^ of June 2023.

The software KNIME 4.6.1 [46] was used to manipulate these tables and to obtain the final dataset for ML analysis. Table DXSUM_PDXCONV_ADNIALL was first filtered to include all patients whose diagnosis changed over time (DXCONV = 1), and then to include patients who specifically converted from MCI to AD (pMCI) (DXCONTYP = 3 and DXCURREN = 3). Table DXSUM_PDXCONV_ADNIALL was then filtered to include patients who did not convert their diagnosis overtime (DXCONV = 0) and who maintained their baseline diagnosis as stable MCI (sMCI) (DXCURR = 2). Here, the column DXCONV was considered as binary variable of the *event* or *censorship* occurrence, i.e., if the event of conversion from MCI to AD occurs its value is 1 (pMCI patient), otherwise is 0 (sMCI patient). The column VISCODE, which reports the number in months of the follow-up visit since the baseline (m06, m12, m18, m24, m36, m48), was used as the *time* variable, or in other words the time of occurrence of the event/censorship [21]. The table ADNIMERGE was joined with the filtered table DXSUM_PDXCONV_ADNIALL, and demographic, clinical, cognitive, CSF and neuroimaging biomarkers of sMCI and pMCI patients at baseline (or at the screening visit according to the assessment) were added from the remaining tables. No further processing of ADNI tables was needed. The implemented KNIME workflow is reported in Additional file 1: Fig. S1. The conversion time interval of MCI patients selected as above ranged from 6 to 48 months (4 years), and all subjects were from ADNI1 protocol. The final dataset consisted of 387 subjects, divided into 216 sMCI and 171 pMCI, and the features were:

- **Demographic variables**: age, gender (PTGENDER), education levels (PTEDUCAT), ethnicity (PTETHCAT) and race (PTRACCAT) [47], marital status (PTMARRY) [48].
- **Biomarker**: APOE4 allele genotype, i.e., presence of APOE gene that makes the ApoE4 protein, associated with late-stage AD [17].
- **Clinical scales**:
  o Clinical Dementia Rating Sum of Boxes (CDRSB), the sum score of the six domains used for accurately stage severity of Alzheimer dementia and mild cognitive impairment [49].
  o Functional Activities Questionnaire (FAQ), an informant-based clinician administered questionnaire that assess the functional daily-living impairment in dementia [50].
- **Neuropsychological assessment**:
  o Alzheimer’s Disease Assessment Scale (ADAS), item 11 and 13, and Delayed Word recall (Q4); for assessing the memory, language, and praxis domains with 11 tasks both subject-completed tests and observer-based assessments [51].
  o Mini-Mental State Examination (MMSE), 30 questions on orientation, short-term memory retention, attention, short-term recall and language to measure cognitive impairment and stage the severity level [52].
  o Rey Auditory Verbal Learning Test (RAVLT), immediate, learning, forgetting and percent forgetting [53].
  o The total delayed recall score of the Logic Memory subtest of the of the Wechsler Memory Scale-Revised (LDELTOTAL), which assesses verbal memory.
  o Digit Symbol Substitution (DIGITSCOR) to evaluate attention, processing speed and executive function.
  o Trails B (TRABSCOR), time to complete part B of the Trail Making Test which assess visual-motor coordination [54].
  o ADNI modified Preclinical Alzheimer's Cognitive Composite (PACC) with Digit Symbol Substitution (mPACCdigit), and with Trails B (mPACCtrailsB) that measure the first signs of cognitive decline [55].
  o Geriatric Depression Scale (GDTOTAL) to identify depression in elderly subjects [12].
  o Total score of Clock Test (COPYSCOR) [12].
  o Boston Naming Test (BNTTOTAL) assesses naming ability using 30 items [12].
- **Cerebrospinal fluid (CSF) biomarker**: Aβ_1–42_ (ABETA42), total tau (TAU), phosphorylated tau (PTAU) concentrations [56].
- **Neuroimaging measures**: MRI volumes of ventricles, hippocampus, whole brain, entorhinal cortex, fusiform, middle temporal gyrus (MidTemp) and total intracranial volume (ICV), calculated with Freesurfer [57]. Average fluorodeoxyglucose positron emission tomography of angular, temporal, and posterior cingulate (FDG) [58]. Hypometabolic convergence index (HCI) [15], an FDG-PET index that provides a single measurement of cerebral hypometabolism compared to AD patients group.

Categorical variables (PTGENDER, PTETHCAT, PTRACCAT, PTMARRY) were converted to numerical data with the One-Hot Encoding approach [20, 59], also called dummy coding (python function *get_dummies()* on Pandas *dataframe*).

### Missing data

ADNI, as well as other international databases, has the problem of missing data, so here, to avoid reducing sample size, we applied the missForest algorithm [60] to impute missing data, which showed better performance than statistical imputation methods on dementia data [61] and on Parkinson's disease data [62]. MissForest is based on RF classification method [4], and it can handle any type of input data (continuous and categorical) by making as few as possible assumptions about the structural aspect of the dataset [60]. In general, missForest uses the mean or the mode to make an initial guess about the missing values before fitting a model using the feature based on the number of missing values, starting with the lowest amount. Missing values are then predicted by using the trained RF and imputed for each feature. Default values of missForest hyperparameters were here used as provided by python package *missingpy* 0.2.0, and the imputation was performed separately on sMCI and pMCI cohorts to maintain the original feature distribution of diagnoses [61].

### Statistical analysis

Differences between patients’ groups in age and years of education were assessed with one-way analysis of variance (ANOVA), differences in distributions of categorical variables were evaluated with Chi-square test, analysis of covariance (ANCOVA) was employed with age and gender as covariates for comparing clinical and cognitive variables, while ANCOVA with age, gender and ICV as covariates for neuroimaging features (significant at *p* < 0.05). All statistical analyses were performed with Python 3.8 and the package *scikit-learn* 1.1.3.

### Survival analysis models

The aim of survival analysis is to assess when an event is likely to happen or to predict the time-to-event such as the time of progression to AD. Survival analysis could handle right-censored data, that is when the event of interest is not observed until the study is terminated, as in the case of stable MCI patients. The waiting time until an event occurs is defined as a positive random variable *T* and given its probability density function *f*(*t*), the cumulative distribution function is:

$${\text{F}}\left( {\text{t}} \right){\text{ = P}}_{{\text{r}}} \left[ {\text{T < t}} \right]{ = }\int\limits_{{{ - }\infty }}^{{\text{t}}} {{\text{f}}\left( {\text{u}} \right){\text{ du}}} .$$

The survival probability *S*(*t*) that the event of interest has not occurred by some time *t* is:

$${\text{S}}\left( {\text{t}} \right){\text{ = 1}}\, - \,{\text{F(t) = P}}_{{\text{r}}} \left[ {{\text{T > t}}} \right].$$

The hazard function *h*(*t*) denotes the approximate probability that an event occurs in the small interval [*t, t* + *dt*), while the cumulative hazard function *H*(*t*) is the integral of the hazard function over the interval [*0;t*]. For discrete time interval subdivided in *J* parts, the risk score of a sample *x* is calculated as:

$${\text{r}}\left( {\text{x}} \right){ = }\sum\nolimits_{{\text{j = 1}}}^{{\text{J}}} {{\text{H (t}}_{{\text{j}}} {\text{, x)}}} .$$

Cox proportional hazard (CPH) [19] is a semi-parametric approach because it makes parametric assumption about the effect of the predictors on the hazard function, but it has no assumptions about the shape of the baseline hazard function, which can take any form. The Cox model is expressed by the hazard function denoted by *h(t),* and it can be estimated as follows:

$${\text{h}}\left( {{\text{t,}}\overrightarrow {{\,{\text{x}}_{{\text{i}}} }} } \right){\text{ = h}}_{{\text{0}}} {\text{(t)}}\eta {\text{(}}\overrightarrow {{{\text{x}}_{{\text{i}}} {\text{}}}} {\text{}}),$$

where *h*_*0*_(*t*) is the unknown baseline hazard function that represents the hazard when all the predictors are equal to zero; $\eta \left( {\overrightarrow {{{\text{x}}_{{\text{i}}} { }}} } \right)$ is the risk function usually defined as a linear representation such as:

$$\eta {( }\overrightarrow {{{\text{x}}_{{\text{i}}} { }}} { }) = {\text{e}}^{{\sum\nolimits_{j = 1}^{p} {x_{j}^{i} w_{j} } }} ,$$

where ${\omega }_{j}$ are the coefficients to determinate and $\overrightarrow{{\text{x}}_{\text{i}}}$ is the observed feature vector.

In CPH, predictors have a multiplicative effect on the hazard function directly. This method uses the partial likelihood to estimate the parameters through partial likelihood function maximization. One of the most important advantages of CPH is the possibility to interpret models like in regression. Despite this, there are some cases such as high data dimensionality and small number of observations, where CPH’s results are unsatisfactory, and it yields incorrect standard deviation for the estimators.

Random Survival Forests (RSF) [27] is an ensemble learner for the analysis of right-censored survival data that follows the same principles of RF for growing decision trees using bootstrapping and random feature selection when splitting tree nodes. The method starts from independent and identically distributed (i.i.d.) random elements:

$$\left( {X,T,\delta } \right),\,\left( {X_{1} ,T_{1} ,\delta_{1} } \right),\,...,\,\left( {X_{n} ,T_{n} ,\delta_{n} } \right),$$

where $X$ is the feature as a *d*-dimensional vector that takes values in a discrete space called χ; *T* = *min(T*^*0*^*, C*^*0*^*)* is the observed survival time defined as the minimum of the true (potentially unobserved) survival event time *T*^*0*^ and the true (potentially unobserved) censoring time C^*0*^; $\delta$ = *1{T*^*0*^ ≤ *C*^*0*^*}* is the binary censoring indicator. When the event conversion-to-AD occurs $\delta$ = *1* (here pMCI), while when the observation is censored $\delta$ = *0* (here sMCI). It is assumed that the true event time *T*^*0*^ is independent of the censoring time *C* [63].

The RSF algorithm is implemented as follow. In the first step, *B* bootstrap samples are selected *ntree* times from the original dataset, leaving approximately one-third of the samples out-of-bags (OOB). A survival tree is grown for each bootstrap sample and *p* candidate variables are randomly selected for each node of each tree. The number *p* is generally the square root of number of independent variables. The node is split when the variable maximizes survival difference between daughter nodes. The splitting rule applied is the log-rank test statistic, calculated to test the null hypothesis that there is no difference between the two groups—here sMCI and pMCI—in the probability of the conversion event. The tree stops to grow if a terminal node has less than the node size unique events. Cumulative hazard functions are calculated for each tree to obtain ensemble’s cumulative hazard estimate. Finally, OOB estimators are used to estimate the prediction accuracy and the variable importance [27, 63].

### Performance evaluation

Survival analysis was conducted with python package *PySurvival* (https://square.github.io/pysurvival/) by Fotso et al. (2019) and its forked repository by Bacalfa (https://github.com/bacalfa/pysurvival/), which adds sklearn compatibility to the CPH and RSF algorithm implementations. Plots were created by modifying the original functions of PySurvival with package *seaborn* 0.12.2.

First, dataset was randomly split with a static seed into training and test sets following the Pareto principle [64] (80–20%, 309–78 patients) stratified by the column *event* to maintain the original distribution of occurrences. Then, optimal values of hyperparameters that maximized the performance on training set were found through a randomized search (RandomizedSearchCV) with threefold cross-validation (*cv*) and 50 repetitions [20, 23]. Hyperparameters of CPH were L2 regularization (l2_reg) and learning rate (lr), while for RSF they were importance mode (importance_mode), maximum depth (max_depth), minimum number of samples required to be at a leaf node (min_node_size), number of features to consider when looking for the best split (max_features) and percentage of original samples used in each tree building (sample_size_pct). The number of trees in RSF was left static and equal to 200, and the initialization method of CPH was ‘zeros’ like in [26].

Performance of ML algorithms was evaluated with the Harrell’s concordance index (*c*-index) [65] both on training set with fivefold cross-validation and on test set. The *c*-index represents a generalization of the area under the ROC curve (AUC) for survival analysis models, which can handle right-censored data, and it estimates the probability that the patients who experienced the event conversion-to-AD first had a worse predicted outcome. Its value provides the model discrimination power, and when it is close to 1, the model has an almost perfect discriminatory power, while if it is close to 0.5 (random prediction), it has no ability to discriminate between low- and high-risk subjects.

The Integrated Brier score (IBS) [66] at time τ was used to evaluate the accuracy of predicted survival function across multiple timepoints on test set. IBS is defined as:

$$IBS\left( \tau \right) = \frac{1}{\tau }\int_{0}^{\tau } {BS\left( t \right)dt} ,$$

where *BS(t)* is the Brier score. IBS is calculated as the average squared distances between the actual survival status and the predicted survival probability, and its value is between 0 and 1, where 0 is for a perfect model, while a cut-off limit of 0.25 is considered as critical [66].

Predicted survival curves by CPH and RSF on test set were compared with the survival curve by Kaplan–Meier (KM) [67], a nonparametric model that is usually applied to visualize the estimated survival time of population. The differences between KM and predicted survival curves were quantified with the root mean square error (RMSE) and median/mean absolute error, as well as visually compared by plotting curves one against other.

### Explainability

#### Global explanation

RSF provides a fully nonparametric measure of variable importance (VIMP), which could be calculated with four different methods: permutation importance [27] and its normalized version [22] that make use of OOB estimation, and impurity and impurity corrected feature importance, which is a bias correction for the Gini index [68]. Here, we automatically selected the optimal VIMP method through hyperparameters tuning [23], as described in the previous section.

Together with the VIMP provided intrinsically by RSF, we computed an external permutation importance, defined as the decrease in model score when a single feature value is randomly shuffled. We applied permutation importance with 50 repetitions provided by ELI5 [42] that has been adapted to the python package scikit-learn 1.3.0.

Shapley Additive Explanation [38] (SHAP) is a model-agnostic unified framework for interpreting ML predictions, and it was here used to investigate further RSF outcomes. SHAP is based on game theory, and it assigns to each feature a Shapley value that represents its average marginal contribution across all possible feature coalitions [39]. A formal definition of SHAP outcome is:

“Prediction *f(x)* for instance *i* differs from the average prediction *E[f(x)]* by *f(x*_*i*_*)- E[f(x)]* to which the feature contributed ${\phi }_{j}^{(i)}$” [33],

where ${\phi }_{j}^{(i)}$ is the SHAP values of the *j*th feature.

SHAP can provide both global explanations—overall feature importance on training set—and local explanations on test predictions. We used the python package *SHAP* 0.42.1, and we built the SHAP explainer (*shap.Explainer*) on RSF predicted risk scores of training set (function *predict_risk* by pysurvival).

The Rank-Biased Overlap (RBO) [43] was used to quantitatively compare the global explanations provided by RSF feature importance, mean permutation importance and mean absolute SHAP (|SHAP|). RBO is a similarity measure between incomplete, top-weighted and indefinite rankings, which has been recently introduced for estimating the overlap between ML feature importance at different depths *d* (number of the top variables considered in the ranking) [35]. RBO assumes values in the range [0, 1], where 0 means disjoint, and 1 means identical. The python package *rbo* (v.0.1.2, https://github.com/changyaochen/rbo) was used as implementation of the RBO by Webber et al. [43].

Although, recent literature showed that in many cases feature selection applied on survival analysis does not provide any improvement in performance [23, 39], we wanted to investigate whether automatic selection of most predictive subset of features could increase RSF performance. With this purpose, we iteratively built RSF models by increasing the number of training features, from the first to the last following their importance order as in the three variable rankings. For each iteration in this recursive feature addition, we evaluated the *c*-index on training set with fivefold cross-validation and on test set.

#### Local explanation

Local explanations on test set were explored with SHAP, and as further analysis we investigated whether correlation between variables could alter the feature contribute to individual risk prediction. With this aim, we used SHAP Partition Explainer, which is a method to handle correlated features by calculating SHAP values based on hierarchical clustering [33]. The first step was to obtain Pearson’s correlation matrix of training set and the second one was to apply hierarchical clustering on the absolute value of correlation coefficients. Clustering results were then provided to SHAP Partition Explainer, and local explanations were visually compared between models with and without correlated features (correlation cutoff =|0.6| as in [26]).

Individual predictions done by RSF were used to manually stratify pMCI test patients according to their conversion-to-AD risk score (low, medium, and high) [26]. Then, one pMCI patient per risk grade was randomly selected (pMCI#1 high risk, pMCI#2 medium risk, pMCI#3 low risk), and their survival probability curve was obtained through estimation of cumulative density function. Finally, we investigated with SHAP waterfall and force plots the local explanations of these three pMCI patients together with one randomly selected stable MCI test subject (sMCI#1) with a numeric risk score lower than 1.

## Results

Demographic, clinical, cognitive, CSF and neuroimaging data of dataset prior to imputation are reported in Table 1 together with missingness percentage and statistical results. sMCI and pMCI groups had significantly different values in almost all features, except for age, gender, education level, RAVLT forgetting, GDTOTAL, COPYSCOR, BNTTOTAL and ICV (*p* > 0.05).

**Table 1:** Demographic, clinical, cognitive, CSF and imaging data of sMCI and pMCI groups

	**sMCI** (216)	**pMCI** (171)	**Missingness** (%)	***p*-value**
Demographic:				
*Age*	74.8 ± 7.4	74.7 ± 6.9	0.0	0.65^a^
*Gender (M/F)*	144/72	105/66	0.0	0.28^b^
*Education level*	15.4 ± 3.1	15.8 ± 2.9	0.0	0.26^a^
Biomarker:				
*APOE4 (0/1/2)*	120/76/20	56/88/27	0.0	** < 0.001**^**b**^
Clinical scale:				
*CDRSB*	1.4 ± 0.8	1.85 ± 0.9	0.0	** < 0.001**^**c**^
*FAQ*	2.4 ± 3.5	5.5 ± 4.9	0.77	** < 0.001**^**c**^
Neuropsychological assessment:				
*ADAS11*	10.4 ± 4.3	13.1 ± 4.1	0.0	** < 0.001**^**c**^
*ADAS13*	16.7 ± 6.2	21.3 ± 5.4	0.77	** < 0.001**^**c**^
*ADASQ4*	5.5 ± 2.2	7.1 ± 1.9	0.0	** < 0.001**^**c**^
*MMSE*	27.3 ± 1.8	26.6 ± 1.7	0.0	** < 0.001**^**c**^
*RAVLT_immediate*	33.6 ± 9.9	27.3 ± 6.4	0.0	** < 0.001**^**c**^
*RAVLT_learning*	3.8 ± 2.5	2.7 ± 2.1	3.36	** < 0.001**^**c**^
*RAVLT_forgetting*	4.5 ± 2.4	4.9 ± 2.1	0.26	0.1^c^
*RAVLT_perc_forgetting*	59.3 ± 33.1	78.7 ± 26.9	0.26	** < 0.001**^**c**^
*LDELTOTAL*	4.6 ± 2.7	3.0 ± 2.7	0.0	** < 0.001**^**c**^
*DIGITSCOR*	38.8 ± 10.8	34.2 ± 10.8	0.26	** < 0.001**^**c**^
*TRABSCOR*	118.5 ± 64.8	147.2 ± 79.3	1.0	** < 0.001**^**c**^
*mPACCdigit*	-6.3 ± 3.1	-8.76 ± 2.8	0.0	** < 0.001**^**c**^
*mPACCtrailsB*	-6.4 ± 3.2	-8.86 ± 3.1	0.0	** < 0.001**^**c**^
*GDTOTAL*	1.54 ± 1.37	1.58 ± 1.36	0.0	0.84^c^
*COPYSCOR*	4.68 ± 0.70	4.58 ± 0.69	0.0	0.15^c^
*BNTTOTAL*	25.86 ± 3.81	25.10 ± 4.24	0.52	0.08^c^
CSF:				
*ABETA42*	981.67 ± 532.47	696.68 ± 358.49	50.39	** < 0.001**^**c**^
*TAU*	293.04 ± 125.75	335.05 ± 113.67	50.39	**0.02**^**c**^
*PTAU*	28.82 ± 14.86	33.47 ± 13.12	50.39	**0.03**^**c**^
Neuroimaging:				
*Ventricles*	42297.1 ± 24293.2	47201.5 ± 23132.8	1.5	**0.002**^**d**^
*Hippocampus*	6722.7 ± 1025.4	6014.1 ± 1020.9	19.1	** < 0.001**^**d**^
*WholeBrain*	1011262.1 ± 104469.7	980,820.5 ± 113713.5	1.3	**0.008**^**c**^
*Entorhinal*	3548.8 ± 731.5	3015.9 ± 713.8	19.1	** < 0.001**^**d**^
*Fusiform*	16894.5 ± 2193.8	15733.7 ± 2471.7	19.1	** < 0.001**^**d**^
*MidTemp*	19597.4 ± 2678.6	17524.6 ± 2980.4	19.1	** < 0.001**^**d**^
*ICV*	1580838.1 ± 164668.7	1571086.5 ± 174884.8	0.0	0.928^c^
*FDG*	1.16 ± 0.15	1.06 ± 0.12	58.91	** < 0.001**^**c**^
*HCI*	7.15 ± 3.50	9.67 ± 3.74	48.32	** < 0.001**^**c**^
Occurrence of event/censorship (sMCI = 0, pMCI = 1) per time point (in months):				
*m06*	20	22		
*m12*	17	47		
*m18*	21	35		
*m24*	31	36		
*m36*	109	27		
*m48*	18	4		

In bold significant result at *p* < 0.05

^a^One-way ANOVA

^b^Chi-square test

^c^ANCOVA with age and gender in covariates

^d^ANCOVA with age, gender and ICV in covariates

Results of hyperparameters tuning obtained through randomized search are reported in Table 2. Optimal hyperparameter values provided a *c*-index (mean of threefold *cv* with 50 repetitions) of 0.798 for CPH and of 0.858 for RSF. Regarding the performance of best models, RSF reached high values of *c*-index both on test set and on training set (0.890, fivefold *cv*: 0.850 ± 0.03), while CPH had lower performance (0.818, fivefold *cv*: 0.766 ± 0.05). IBS score was the same for RSF and CPH (0.09).

**Table 2:** Hyperparameters of Cox proportional hazard (CPH) and Random Survival Forests (RSF)

	Hyperparameter	Parameter distribution	Optimal value
CPH	*l2_reg*	float from a *reciprocal continuous random distribution* in range (0.1, 100)	99.4
	*lr*	float from a *reciprocal continuous random distribution* in range (0.1, 1)	0.79
RSF	*importance_mode*	['normalized_permutation','permutation','impurity','impurity_corrected']	‘permutation'
	*max_depth*	integer from a *reciprocal continuous random distribution* in range (5, 50)	24
	*min_node_size*	integer from a *reciprocal continuous random distribution* in range (1, 40)	7
	*max_features*	[‘all’,'sqrt','log2']	‘sqrt’
	*sample_size_pct*	[0.60,0.70,0.80,0.90]	0.80

Tuning was performed through a randomized search with threefold cross-validation and 50 repetitions

**Fig. 1:**
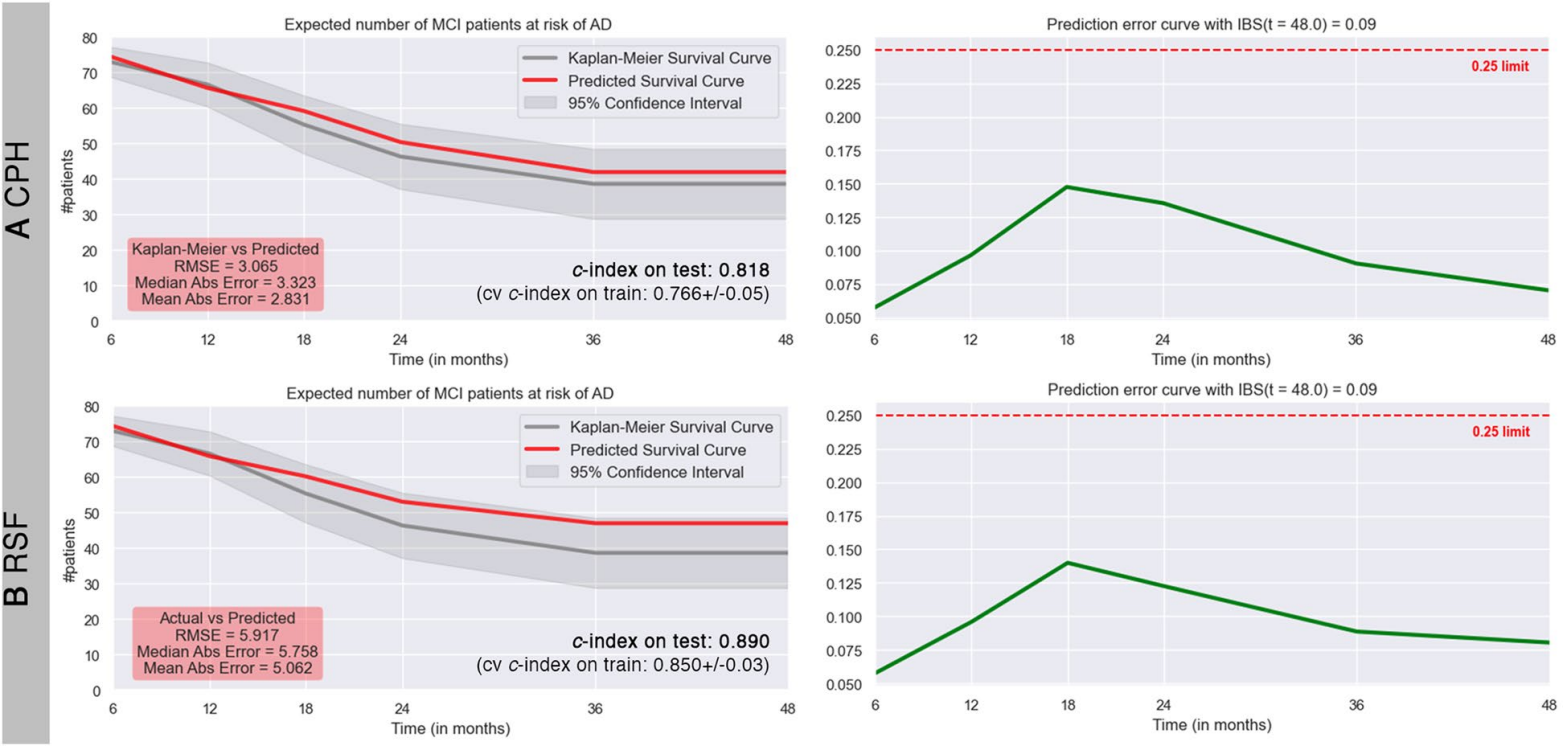
Performance on test set of ML survival algorithms per timepoint: **A** Cox proportional hazard (CPH), **B** Random Survival Forests (RSF). On the left: plots over time of expected number of MCI patients at risk of conversion to AD, predicted survival curve in red, estimated survival curve by Kaplan–Meier in gray. On the right: prediction error curve calculated with Integrated Brier Score (IBS, critical cut-off limit of 0.25 in red). *C*-index on test set, cross-validated (*cv*) *c*-index on training set (mean ± standard deviation), root mean square error (RMSE) and median and mean absolute error are also reported

It could be noted from plots comparing KM and predicted survival curves (Fig. 1 on the left) that accuracy of CPH and RSF models decreased while time progresses. In other words, predicted number of MCI patients at risk of AD differs more from KM estimate as timespan reaches 48 months after baseline visit. In the comparison between KM curve and predicted curves, although both CPH and RSF were close to the actual one, CPH had lower RMSE, median and mean absolute error than RSF, which anyway relies in the 95% confidence interval of KM estimate (Fig. 1 A and B on the left).

Regarding the prediction error per each timepoint (Fig. 1 A and B on the right), CPH and RSF never exceeded the IBS cut-off (dotted red line), although they both showed a global maximum at the 18th month.

**Fig. 2:**
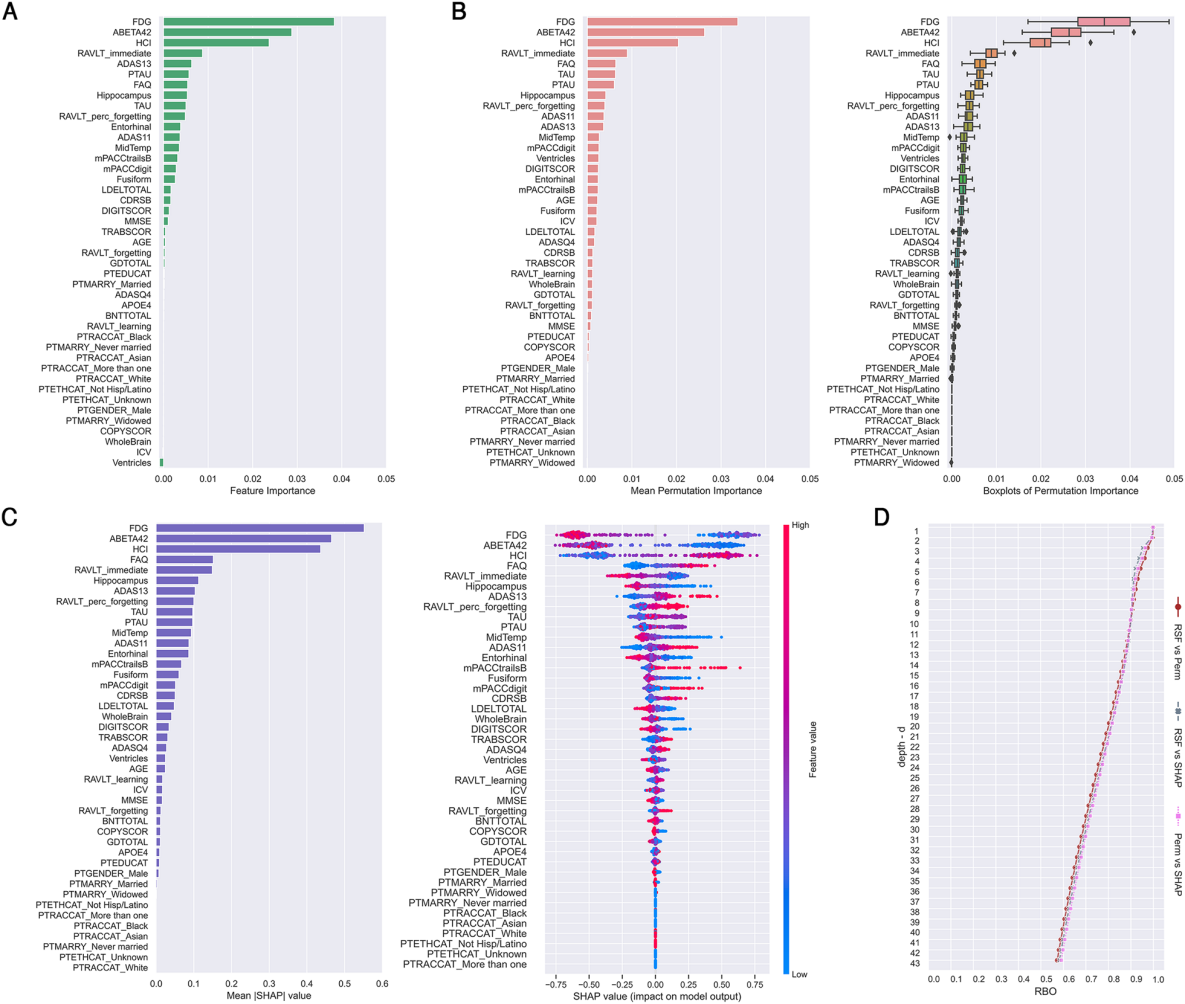
Global explanations of Random Survival Forests (RSF). **A** RSF feature importance (VIMP). **B** Permutation importance (mean value and boxplots). **C** SHAP importance (mean |SHAP| value and SHAP value as beeswarm plot). **D** Rank-Biased Overlap (RBO) curves of variable rankings comparison for increasing values of depth *d* (number of important features considered) between RSF feature importance and mean permutation importance (in brown), RSF feature importance and mean |SHAP| importance (in gray), mean permutation importance and mean |SHAP| importance (in pink)

Global explanations on training set are reported in Fig. 2, where RSF feature importance (Fig. 2A), permutation importance (Fig. 2B, mean value and boxplots) and SHAP importance (Fig. 2C, mean absolute value and beeswarm plot) are depicted as ranking of features ordered by their prediction importance. In SHAP beeswarm plot, one point corresponds to a single patient, where its position along the *x* axis provides the impact that a feature had on the model’s output. In the present work, the feature impact corresponds to the contribute to conversion-to-AD risk, that is a patient with higher SHAP value has higher risk to progress to AD relative to a patient with lower SHAP value.

Top three features FDG, ABETA42 and HCI were in identical order across the three rankings (Fig. 2A–C). RBO curves of similarity between rankings by raising depth *d* are depicted in Fig. 2D (RSF vs Perm in brown, RSF vs SHAP in gray, Perm vs SHAP in pink). All three pairwise comparisons showed an RBO > 0.90 within 8 top variables, with a percentage overlap between RSF importance and permutation importance of 90.7%, between RSF importance and SHAP importance of 90.4%, and between permutation importance and SHAP importance of 90.3%.

Regarding the feature selection, no performance improvement on training or test set was found, in fact subsets of ranked variables worsened RSF *c*-index, and consequently we did not report any results.

**Fig. 3:**
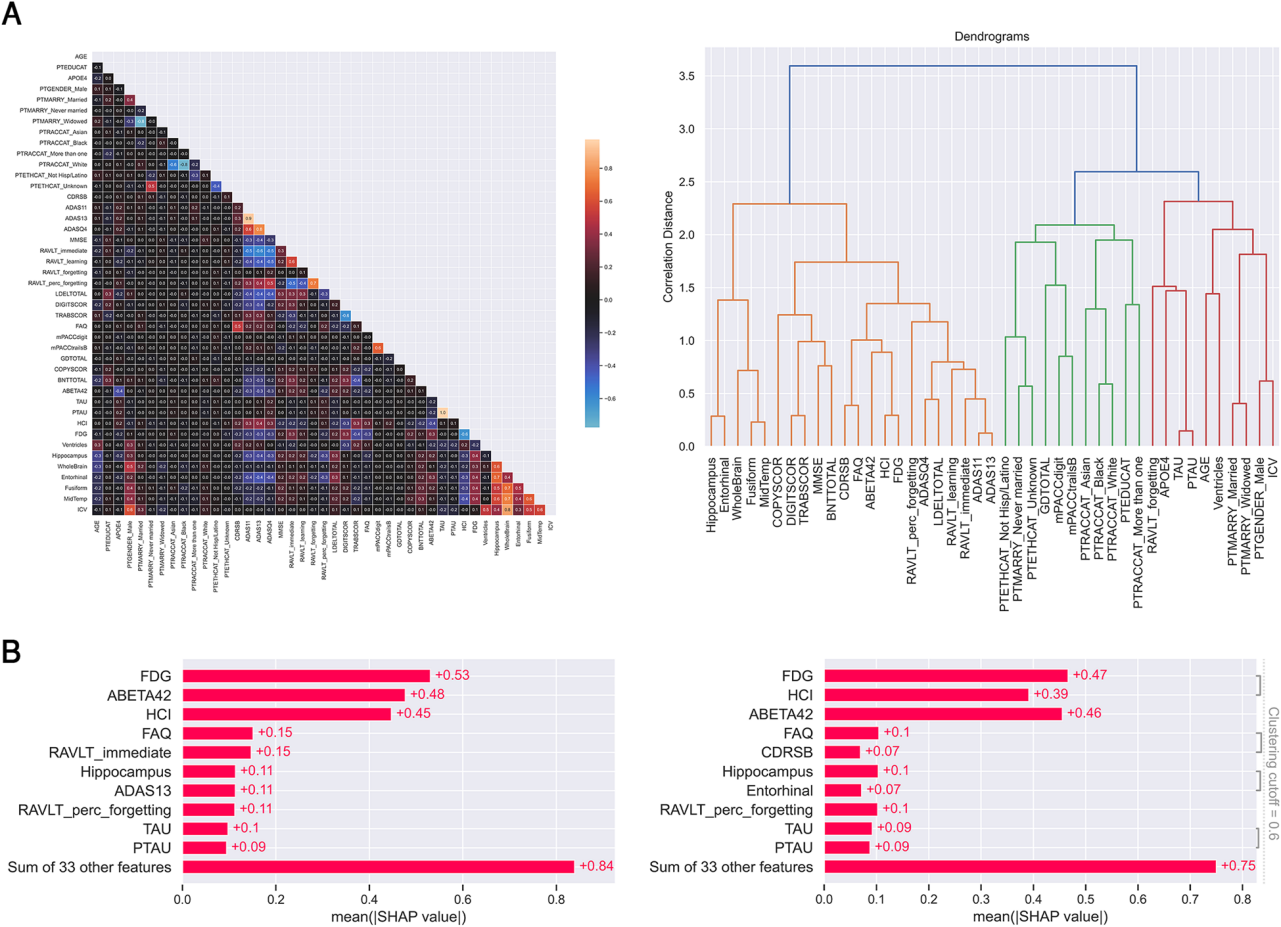
Results of variables correlation analysis. **A** Pearson’s correlation matrix of training set (on the left) and dendrogram (on the right) of hierarchical clustering on absolute value of correlation coefficients. **B** Comparison of mean |SHAP| values on test set, i.e., local explanations, of models with (on the left) and without (on the right) correlated features (clustering cutoff 0.6)

Findings about the impact of variables correlation on local explanations are depicted in Fig. 3. Correlation matrix is on the left of Fig. 3A, while dendrogram of hierarchical clustering is on the right.

Comparison of SHAP local explanations in Fig. 3B showed that features that most contributed to risk prediction did not differ between models with and without correlated variables and they were almost in identical order. Moreover, Fig. 3B confirmed global explanations (Fig. 2), where the highest contribute was provided by FDG, ABETA42 and HCI.

**Fig. 4:**
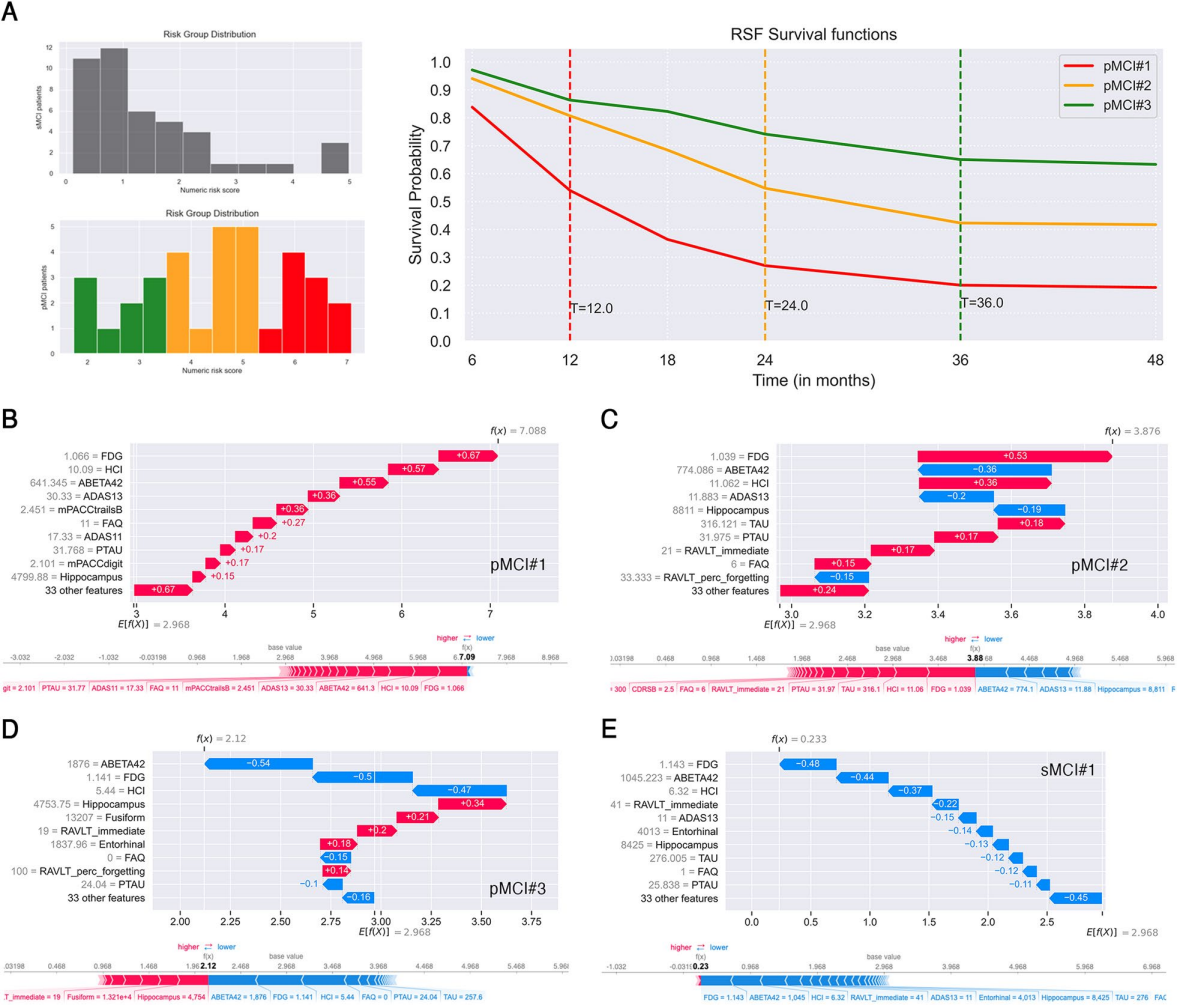
Local explanations of Random Survival Forests (RSF). **A.** On the left: histograms of sMCI and pMCI patients’ risk distribution predicted by RSF. pMCI subjects were stratified by risk grade: low (in green, between 1 and 3.5), medium (in orange, between 3.5 and 5), high (in red, between 5 and 7.1). On the right: RSF survival functions of pMCI patients per risk score: pMCI#1 high risk (score 7.088, converted to AD after 12 months), pMCI#2 medium risk (score 3.876, converted to AD after 24 months), pMCI#3 low risk (score 2.12, converted to AD after 36 months). SHAP waterfall plot (top) and force plot (bottom) of **B.** patient pMCI#1, **C.** patient pMCI#2, **D.** patient pMCI#3, **E.** stable MCI patient who does not convert to AD within 48 months (sMCI#1, risk score 0.233). Blue and red arrows represent those features that, respectively, decrease and increase the conversion-to-AD risk within 48 months. Average predicted risk *E[f(x)]* = 2.968. Actual value of feature in gray

Histograms of conversion-to-AD risk score distribution predicted by RSF on sMCI and pMCI test subjects are on the left of Fig. 4A. Twenty-two sMCI patients had a predicted risk score lower than 1, eighteen subjects had a risk score between 1 and 4, while three patients had a predicted risk score higher than 4.

Manual stratification of pMCI was performed by grouping patients according to three risk grades: low between 1 and 3.5 (in green), medium between 3.5 and 5 (in orange), high between 5 and 7.1 (in red). RSF survival functions of three randomly selected pMCI subjects per risk grade are depicted on the right of Fig. 4A. High risk patient pMCI#1 had a risk score of 7.088, converted to AD at the 12th month* and predicted survival probabilities at each time point were [0.84, 0.54*, 0.36, 0.27, 0.20, 0.19]. Medium risk patient pMCI#2 had a risk score of 3.876, converted to AD at the 24th month*, and predicted survival probabilities at each time point were [0.94, 0.81, 0.68, 0.55*, 0.42, 0.41]. Low-risk patient pMCI#3 had a risk score of 2.12, converted to AD at the 36th month*, and predicted survival probabilities at each time point were [0.97, 0.863, 0.82, 0.74, 0.65*, 0.63]. The drop in the predicted survival probability has been highlighted in the text with an asterisk, and in other words we can state that pMCI#1 had a low probability to remain stable at the 12th month (54%), pMCI#2 had a low probability to remain stable at the 24th month (55%), and pMCI#3 had a low probability to remain stable at the 36th month (65%). From the RSF survival functions in Fig. 4B, it could be further noted that the first sudden drop in survival probability curve corresponds exactly to the actual time of conversion for all the three pMCI test patients, demonstrating that RSF predicted accurately their conversion-to-AD risk. sMCI#1 subject—who does not convert to AD within 48 months—had risk score 0.233 and very high predicted survival probabilities per time point [0.99, 0.98, 0.98, 0.97, 0.95, 0.94].

SHAP waterfall and force plots of pMCI#1, pMCI#2, pMCI#3, and sMCI#1 patients are reported, respectively, in Fig. 4B–E; average predicted risk was *E[f(x)]* = 2.968, and actual value of each feature is also reported (in gray). Arrows show the influence of each variable on risk prediction: blue arrow indicates that the feature decreases the risk of conversion from MCI to AD, while red arrow indicates that the feature increases it. The combined effects of all features provide the final SHAP value, which corresponds to the prediction risk score. It is worth of noting that we used SHAP explainer built on all features, since the comparison between models with and without correlated variables showed no substantial difference in local explanation.

Variables with the highest influence on risk prediction of pMCI#1, pMCI#2, pMCI#3 and sMCI#1 subjects were FDG, ABETA42 and HCI (Fig. 4B–E), as also found in global and local explanation (Figs. 2A–C, 3B).

Local explanations of the three sMCI test subjects with a predicted numeric risk score higher than 4 (Fig. 4A, histogram of sMCI risk distribution in gray) were reported in Additional file 1. Considering that in these three sMCI patients (sMCI#2, sMCI#3, sMCI#4) the conversion did not occur within 4 years, it is interesting to understand why on the contrary, RSF predicted a medium risk score. The predicted survival probability of patients sMCI#2, sMCI#3 and sMCI#4 showed a sudden drop at the 18^th^ month, with, respectively, 58%, 57% and 63% of probability to remain stable. Their waterfall and force plots are reported in (Additional file 1: Fig. S2), and the first three features contributing to increase the conversion-to-AD risk were FDG, ABETA42 and HCI.

## Discussion

The main aim of the present work was to provide a comprehensive overview of the explainability of Random Survival Forests (RSF) in predicting the conversion-to-AD risk within 4 years. We applied RSF on data from ADNI, which consisted in clinical, cognitive, CSF and neuroimaging biomarkers of stable and progressive MCI patients.

Our findings confirmed that RSF improves the prediction power of traditional survival method CPH on dementia data [20, 21, 26, 30, 44, 45]. Our RSF accuracy (0.89) was higher than in similar studies on AD progression, 0.75 by Nakagawa et al. [44], 0.831 by Mirabnahrazam et al. [45], 0.84 by Orozco-Sanchez et al. [21] and by Musto et al. [30], 0.86 by Song et al. [31], and 0.87 in our recent work by Sarica et al. [26]. Spooner et al. [20] performed better (0.93) probably because they used also longitudinal data rather than baseline data alone as in the present and other works.

In addition to the recent literature, we performed for the first time a comprehensive study on RSF explanations. First, we investigated the RSF global explanations by quantitatively comparing with RBO three different feature rankings, the intrinsically provided VIMP, the permutation importance and the SHAP importance. The percentage pairwise overlap between variable rankings was higher than 90% within the first eight features, showing the stability and robustness of RSF also in presence of multicollinearity among features. The stability of RSF algorithm against multicollinearity was also demonstrated by our comparison of SHAP explainers with and without correlated features, which had no substantial difference in local explanations.

Interestingly, the first three important features FDG, ABETA42 and HCI were the same not only in the three global explanations on training set, but also in the local explanation of test set. The feature FDG is the average counting of angular, temporal, and posterior cingulate regions [52] and it is considered as an independent biomarker for AD diagnosis, as demonstrated in a longitudinal study by Ou et al. [69]. Abnormal FDG-PET were found in the 72.82% of pMCI [69], suggesting that subjects with low glucose metabolism have a higher risk to progress to AD as in the present work, where FDG had the highest mean |SHAP| value in local explanation (+ 0.53, Fig. 3B). ABETA42 is considered the CSF biomarker signature of AD and the most sensitive biomarker for AD compared with TAU and PTAU [50]. Hansson et al. [70] demonstrated that MCI patients had an increase in the relative risk of progression to AD in presence of pathological concentrations of T-tau and Aβ42 at baseline. In other words, an increment in levels of CSF tau associated with a decline in levels of CSF Aβ1-42 may indicate the onset of AD before the manifestation of clinical symptoms [50]. In our findings ABETA42 was the second most important feature (+ 0.48, Fig. 3B), while TAU was among the first ten features in the three global explanations as well as in the local explanation (+ 0.1, Fig. 3B). The third most important feature was HCI (+ 0.45, Fig. 3B), which is a hypometabolic convergence index introduced to assess FDG-PET hypometabolism in dementia patients with a single measurement [15]. It has been shown by Chen et al. [15] that MCI patients with high HCI values or low hippocampal volumes had the highest hazard ratios in progressing to AD within 18 months, and those with both characteristics had a much higher risk. In our investigation, we confirmed the importance of hippocampus volume as one of the first eight features that contributed most to the increase in conversion-to-AD risk score (+ 0.11, Fig. 3B).

FAQ, mPACCdigit, mPACCtrailsB and RAVLT immediate had the highest mean |SHAP| values among all clinical and neuropsychological assessments. Their importance in the prognosis of AD was already demonstrated in our previous work on tree-based ML survival methods by Sarica et al. [26] and in Spooner et al. [20]. FAQ is a collateral-report scale that evaluates instrumental activities of daily living [50], and it can differentiate MCI from AD given that functional changes are found early in dementia patients. In particular, Teng et al. [71] demonstrated the prognosis utility of FAQ, showing that it exhibits optimal accuracy (84.7%), sensitivity (80.3%) and specificity (87.0%) in discriminating MCI patients from very mild AD patients. In the present study, FAQ was the most important clinical scale in local explanation on test set (mean |SHAP| value + 0.15, Fig. 3B), and among the first eight features in global explanations (Fig. 2). The mPACCdigit and mPACCtrailsB tests measure, respectively, working memory and performance of processing speed [55], while RAVLT immediate assesses the total acquisition/learning in episodic memory [53]. The contribute of mPACCtrailsB (+ 0.36), FAQ (+ 0.27) and mPACCdigit (+ 0.17) in the increment of conversion-to-AD risk score is particularly evident in the local explanation of patient pMCI#1 (Fig. 4B), who had a numeric risk score of 7.09 and converted at the 12th month after the baseline diagnosis. Interestingly, the explanations of patient sMCI#1, who do not convert to AD within 4 years, showed that RAVLT immediate was the cognitive feature that most contributed to reduce the risk of progression to AD (− 0.22, Fig. 4E).

It is worth of noting that, as in two works on survival ML methods [23, 39], we found that feature selection by recursive feature elimination on the three importance rankings investigated, did not provide any improvement in the overall performance. This is probably due to the feature selection internally performed by RSF to handle high-dimensional data [20], or as hypothesized by Jung et al. [23], better results could be obtained with feature selection methods specially designed for right-censored data.

Our work has three limitations related to the ADNI dataset used for the analysis. The first issue is linked to the variable *time*, which had imbalanced distribution of event/censorship occurrences per timepoint. Indeed, we cannot exclude that the global and local explanations were biased toward the characteristics of the majority class (sMCI). At the present time, no works exist about the stability of RSF outcome on imbalanced groups, thus we cannot exclude that variable rankings may change with better balanced datasets. The other limitation regards more strictly the RSF performance, which may be improved by adding longitudinal data of MCI patients, as in Spooner et al. [20], although such a choice is hardly applicable on datasets with high missingness percentage as in ADNI. The last issue related to the dataset is that dementia conversion diagnosis and its estimate of time of occurrence are prone to human errors, and such errors inevitably introduce bias in survival algorithms, as demonstrated by the three sMCI patients who were incorrectly predicted by RSF with a medium-risk score and a low survival probability (Additional file 1: Fig: S2).

Regarding our methodology, it should be reported that we used a randomized search for hyperparameters tuning, which is not an exhaustive search and thus it is possible that better accuracy can be reached with hyperparameter values not here applied. Another limitation related to our methodology is the application of SHAP post hoc explanation method on survival models. Although it was successfully employed in other ML survival studies [39–41], we must highlight that SHAP was born to explain supervised classification problems. Indeed, SHAP has been adapted for survival models by using single-point risk predictions, as in the present study, or by aggregating survival functions [39], and doing so the information contained in the survival distribution could be lost [72]. Future works are needed to investigate RSF time-dependent explanations in predicting conversion-to-AD risk with model-agnostic methods specifically designed for survival analysis, such as survLIME [73] or survSHAP [72], which have been very recently introduced.

## Conclusion

In summary, we provided a comprehensive study about the explainability of RSF in predicting conversion-to-AD risk on data from ADNI, comprising demographic, clinical, genetic, CSF and neuroimaging biomarkers. We found that RSF improved the performance of the traditional survival method CPH. The stability and robustness of RSF algorithm was highlighted through a quantitative comparison of three different feature importance rankings, the VIMP intrinsically provided by RSF, the permutation importance and SHAP importance, which showed a high percentage of similarity (> 90%) within the first eight features. Most importantly, we demonstrated that multicollinearity among variables does not perturb the local explanations of RSF. Another important contribution of the present work is that we found that feature selection does not improve the RSF performance on training and test sets. Finally, the local explanations of individual pMCI patients gave important information about the contribution of each feature in the conversion-to-AD risk score.

Taken together, our findings suggest that ML algorithms for survival analysis, and in particular RSF method, represent a useful tool to support clinicians in the assessment of conversion-to-AD risk, especially when high-dimensional and heterogenous data are employed. Moreover, the application of SHAP explainer boosts the clinical utility of such approaches, providing intelligible and interpretable plots, which highlight the key features associated with the AD progression also at individual level.

### Supplementary Information

**Additional file 1: Fig S1.** KNIME 4.6.1 workflow implemented to manipulate csv tables from ADNI. **Fig S2.** Local explanations of Random Survival Forests (RSF) on the three sMCI with medium-risk predicted score > 4. A. Patient sMCI#2 with predicted risk score 4.98 and predicted survival probabilities per time point [0.90, 0.73, 0.58, 0.44, 0.30, 0.29]. B. Patient sMCI#3 with predicted risk score 4.95 and predicted survival probabilities per time point [0.91, 0.70, 0.57, 0.46, 0.32, 0.31]. C. Patient sMCI#4 with predicted risk score 4.60 and predicted survival probabilities per time point [0.93, 0.78, 0.63, 0.48, 0.33, 0.31]. Blue and red arrows represent those features that, respectively, decrease and increase the conversion-to-AD risk within 48 months. Average predicted risk E[f(x)] = 2.968. Actual value of feature in gray.

## Data Availability

The data sets used and/or analyzed during the current study, the KNIME workflow and python source code are available from the corresponding author on reasonable request.

## Abbreviations

AD: Alzheimer’s disease
CPH: Cox proportional hazard
CV: Cross-validation
IBS: Integrated Brier Score
KM: Kaplan–Meier
MCI: Mild cognitive impairment
pMCI: Progressive MCI
RBO: Rank-Biased Overlap
RF: Random forest
RSF: Random Survival Forests
SHAP: SHapley additive exPlanations
sMCI: Stable MCI
VIMP: Variable importance
