# Supplementary material for: Explainability of random survival forests in predicting conversion risk from mild cognitive impairment to Alzheimer’s disease
Source: Brain Inform. 2023 Nov 18;10(1):31. doi: 10.1186/s40708-023-00211-w (PMC10657350; doi:10.1186/s40708-023-00211-w)
Supplement: Supplementary file 1 — Additional file 1: Fig S1. KNIME 4.6.1 workflow implemented to manipulate csv tables from ADNI. Fig S2. Local explanations of Random Survival Forests (RSF) on the three sMCI with medium-risk predicted score > 4. A. Patient sMCI#2 with predicted risk score 4.98 and predicted survival probabilities per time point [0.90, 0.73, 0.58, 0.44, 0.30, 0.29]. B. Patient sMCI#3 with predicted risk score 4.95 and predicted survival probabilities per time point [0.91, 0.70, 0.57, 0.46, 0.32, 0.31]. C. Patient sMCI#4 with predicted risk score 4.60 and predicted survival probabilities per time point [0.93, 0.78, 0.63, 0.48, 0.33, 0.31]. Blue and red arrows represent those features that, respectively, decrease and increase the conversion-to-AD risk within 48 months. Average predicted risk E[f(x)] = 2.968. Actual value of feature in gray. [file 40708_2023_211_MOESM1_ESM.docx]

**SUPPLEMENTARY MATERIAL**

**Explainability of Random Survival Forests in predicting conversion risk from Mild Cognitive Impairment to Alzheimer’s disease**


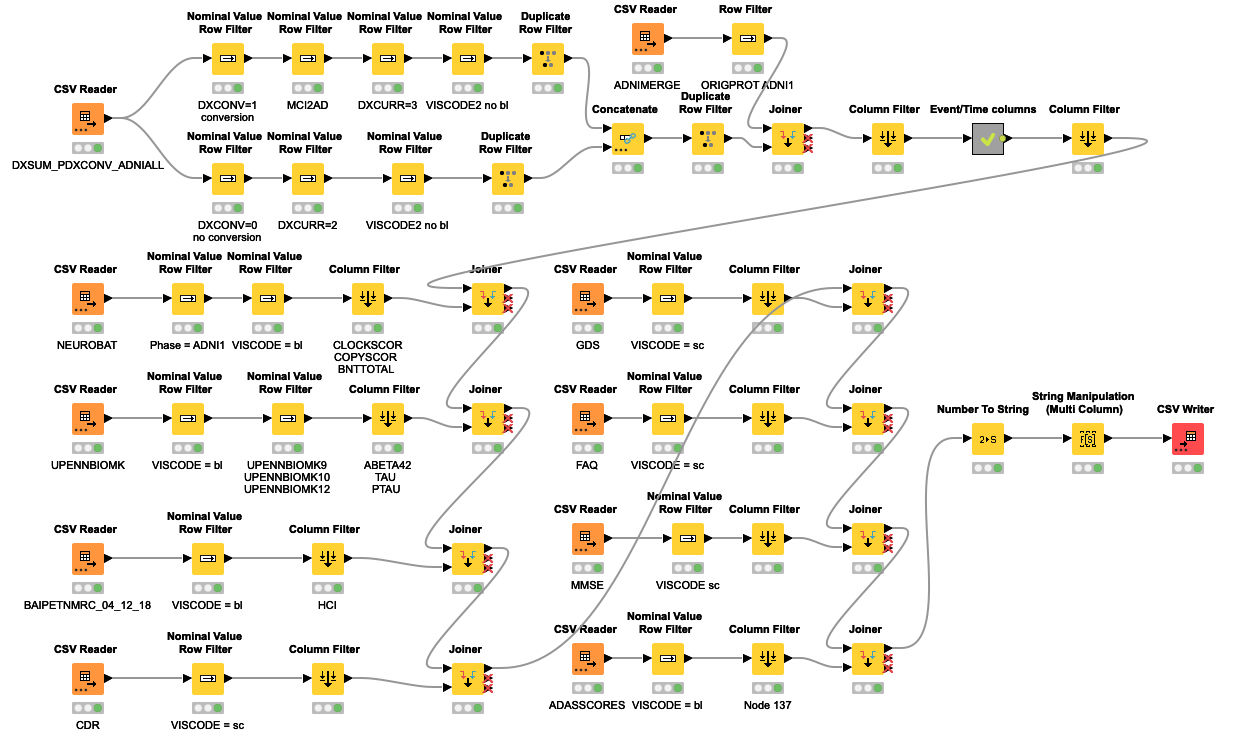


**Fig. S1.** KNIME 4.6.1 workflow implemented to manipulate csv tables from ADNI.


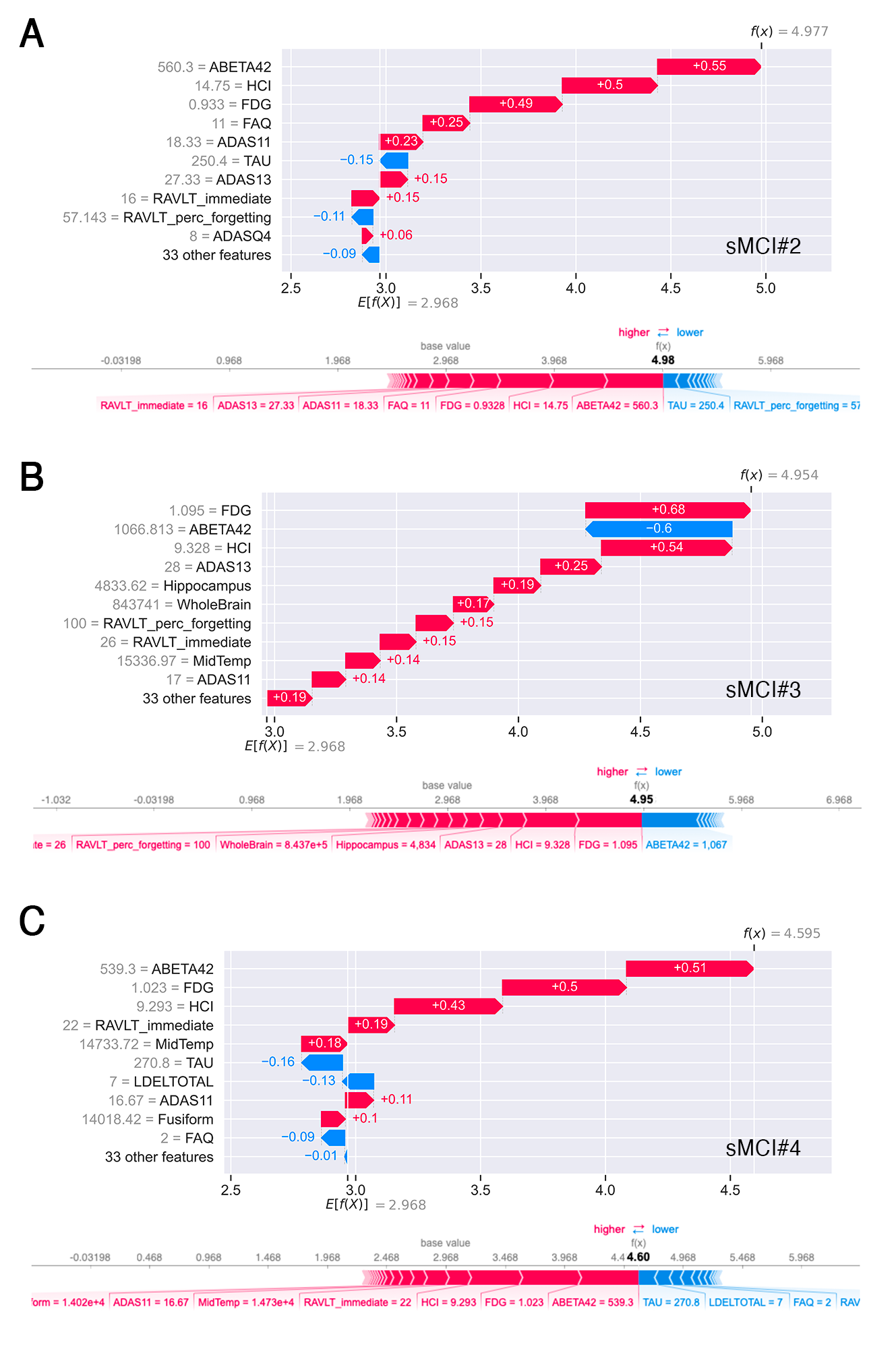


**Fig. S2**. Local explanations of Random Survival Forests (RSF) on the three sMCI with medium-risk predicted score > 4. **A.** Patient sMCI#2 with predicted risk score 4.98 and predicted survival probabilities per time point [0.90, 0.73, 0.58, 0.44, 0.30, 0.29]. **B.** Patient sMCI#3 with predicted risk score 4.95 and predicted survival probabilities per time point [0.91, 0.70, 0.57, 0.46, 0.32, 0.31]. **C.** Patient sMCI#4 with predicted risk score 4.60 and predicted survival probabilities per time point [0.93, 0.78, 0.63, 0.48, 0.33, 0.31]. Blue and red arrows represent those features that respectively decrease and increase the conversion-to-AD risk within 48 months. Average predicted risk *E[f(x)]* = 2.968. Actual value of feature in gray.
